# Piezoelectric line detector array for photoacoustic tomography

**DOI:** 10.1016/j.pacs.2017.09.002

**Published:** 2017-09-21

**Authors:** Guenther Paltauf, Petra Hartmair, Georgi Kovachev, Robert Nuster

**Affiliations:** Department of Physics, Karl-Franzens-Universitaet Graz, Universitaetsplatz 5, 8010 Graz, Austria

**Keywords:** Optoacoustic, Thermoacoustic, Tomography, Piezoelectric polymer, Ultrasound array

## Abstract

Photoacoustic tomography relies on a dense coverage of the surface surrounding the imaged object with ultrasound sensors in order to enable an accurate reconstruction. A curved arrangement of integrating line sensors is proposed that is able to acquire data for a linear projection image of the absorbed energy density distribution in the object. Upon rotation of the object relative to the array, three-dimensional (3D) images can be obtained.

The proposed design is based on the cost-effective piezoelectric polymer film technology with 64 line shaped sensors arranged on a half-cylindrical surface. It is combined with an optical parametric oscillator for the near infrared as a source for laser pulses. Image reconstruction from recorded signals consists of two-dimensional (2D) back projection followed by an inverse Radon transform.

The tomograph exhibits a spatial resolution on the order of 200 to 250 μm. In a phantom experiment, the steps from acquisition of a single, 2D projection image to a full 3D image are demonstrated. Finally, in vivo projection images of a human finger are shown, revealing the near real-time imaging capability of the device in 2D.

## 1 Introduction

Photoacoustic (or optoacoustic) imaging has been established as a method for obtaining images of biological tissue with optical contrast and high resolution. The combination of these favorable properties arises from the thermoelastic generation of sound waves by deeply penetrating, diffuse light from pulsed laser sources. In three-dimensional (3D) photoacoustic tomography (PAT), sensors are distributed around the volume of interest to detect the sound waves. PAT devices are composed of optical and ultrasound components and may become quite complex. Although still much less expensive than magnetic resonance tomography devices, the costs of commercial photoacoustic tomographs for small animal imaging are rather high and it has become an attractive alternative to design and manufacture PAT devices in the own lab. This is facilitated by the availability of multichannel analog to digital converters with the desired bandwidth. Also, pulsed laser sources that are suitable for photoacoustic excitation, primarily optical parametric oscillators (OPOs), are offered with specifications ideal for this purpose.

**Fig. 1:**
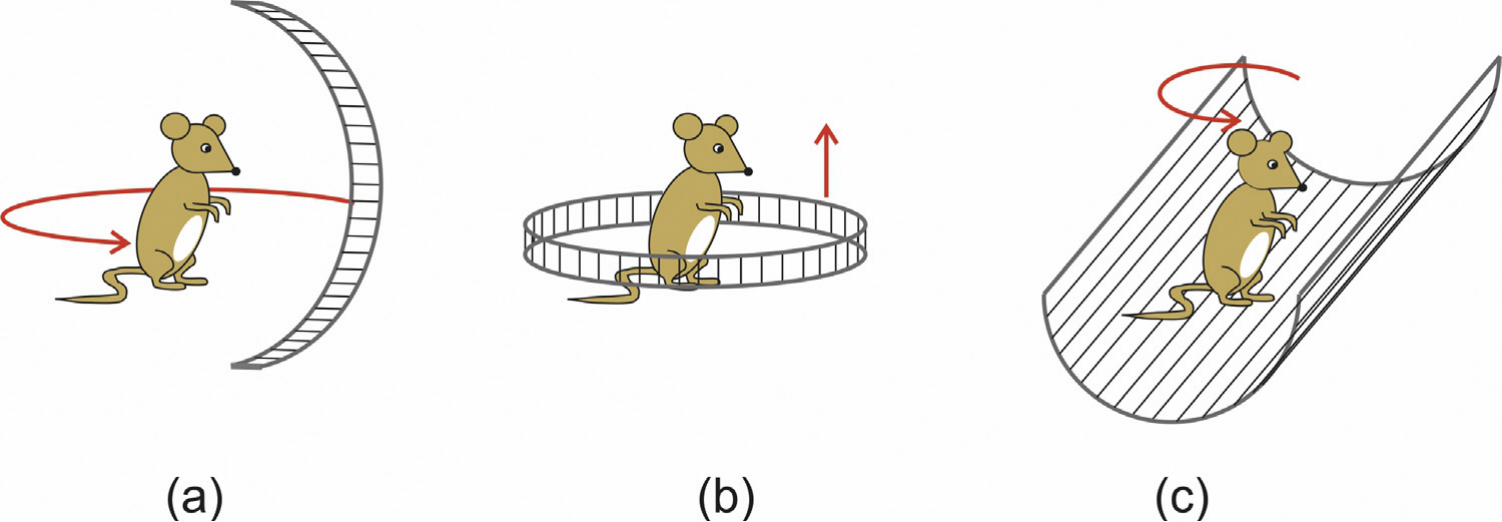
Detection geometries for 3D photoacoustic tomography with a scanning array. (a) Curved array emulating a spherical array. (b) Ring shaped array for scanning an object layer by layer. (c) Line detector array generating 3D images from projections.

In PAT, images of the absorbed energy density are reconstructed by applying appropriate tomographic algorithms to the acquired time-resolved signals. For an accurate reconstruction, a dense coverage of a preferably closed detection surface with sensor elements is crucial. This can require several hundred to tens of thousands detector positions. To achieve this high number of detectors, several approaches have been proposed. Very flexible is the scanning of a single sensor across an area surrounding the object or a plane close to the object [1], [2]. However, this leads to a data acquisition time that is determined by the pulse repetition rate of the excitation laser and ranges from several minutes to hours. Therefore, several solutions involving sensor arrays have been developed. A recent development is a curved array covered by 512 elements, which allows single-shot, 3D tomographic images and has proven very useful for dynamic studies [3]. To achieve an even higher number of detectors on the order of several times 10^3^ at a low level of complexity that allows the devices to be home-made, it is possible to use a relatively small array of sensors arranged on a curve or line that is moved relative to the object. Fig. 1 shows implementations of such moving arrays. A curved array rotating relative to the object (Fig. 1a) emulates a spherical array with thousands of virtual detector points [4]. With this array, an image can be reconstructed by applying a 3D algorithm to the data. A 3D image composed of a series of 2D sectional images is generated by a ring shaped array (Fig. 1b) that encloses the object and is translated along the ring axis [5], [6]. Each of the 2D slices can be viewed as a stand-alone image and dynamic processes within the slice can be observed in real time. In this work, we present an array of extended, line shaped detectors (Fig. 1c), which generates 2D projections of the photoacoustic sources [2]. A 3D image from projections requires a rotation of the object relative to the array followed by a second tomographic reconstruction. In each projection image, always the whole imaging field is visible, contrary to a section image, which shows only a single slice at a time. This feature is comparable to x-ray projection imaging and can be used for observing dynamic processes in a large volume or in cases where the exact location of the region of interest is not a priori known.

Line detectors have special features that distinguish them from point-like detectors. The first one is the reduction of the imaging problem to two dimensions, leading to the projection imaging capability described above. Another, favorable property is the geometrical attenuation. While point like detectors receiving signals from a small source will show a drop of signal amplitude proportional to the reciprocal distance, line detectors only exhibit an attenuation proportional to the inverse of the square root of the distance. Furthermore, if detectors with a finite size are treated as point detectors in image reconstruction, this leads to a limitation in image resolution [7]. For line detectors it could be shown that their linear extension, if it sufficiently exceeds the dimension of the imaged object, has no effect on the image resolution, since it is naturally taken into account in the 2D image reconstruction algorithm [8]. Line sensors can be very efficiently fabricated by employing optical techniques, since a light beam, either in free space or in an optical waveguide, is a natural line shaped receiver. First implementations have therefore used optical interferometric sensors [2], [9], [10]. In addition, non-interferometric methods, such as beam deflection, can be used as optical line detectors [11], [12]. Photoacoustic tomographs based on interferometric sensors have mostly used single element scanning, limiting their data acquisition speed considerably. Therefore, there have been several attempts to enable optical array detection, such as in a recent work demonstrating an array of fiber optic sensors [13]. In another recent study, a CCD camera was used to take snapshots of the projection of photoacoustically generated waves along a collimated probe beam in an optical phase contrast setup [14]. The spatial wave pattern has equal information content as a linear arrangement of line sensors detecting temporal signals. Despite their obvious benefits, the integration of optical sensors in arrays is still very demanding and requires high technological efforts. By contrast, piezoelectric transducers are a technique that more easily can be implemented in a compact, home-made tomographic device.

In this work, we present a photoacoustic tomograph manufactured of relatively low-cost components such as standard electronics and a piezoelectric polymer film, combined with an OPO for the near infrared. The tomograph has 64 line sensors in an arrangement as shown in Fig. 1c, where the elements are located on a semi-cylindrical surface. The curved shape of the array resulted from considerations regarding the limited-view problem in tomographic reconstruction: All boundaries of an object can be resolved with maximum resolution if signals are recorded from at least 180° around the object [15], [16]. Due to parallel detection of the line detector signals, the array is capable of acquiring almost real-time, 2D projection images of the distribution of energy density in an object after absorption of a laser pulse. In the following, first the design of the tomograph is shown and the steps in manufacturing the device. Then, an imaging experiment is presented, where the steps of 3D image generation are demonstrated on a phantom. This is followed by several experiments for its characterization in terms of resolution, sensitivity and bandwidth. Finally, the projection imaging capabilities of the device are shown in an in vivo experiment.

## 2 Methods

### 2.1 Design of the tomograph

**Fig. 2:**
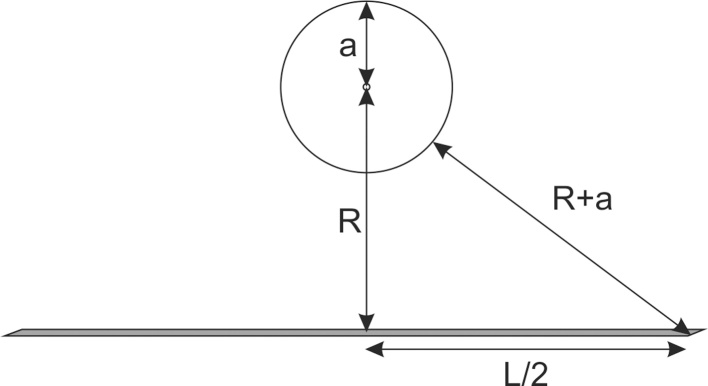
Illustration of the required line length condition for objects contained in a sphere with radius *a* at distance *R* from the line detector.

The tomograph was designed following the requirements for an array of line shaped sensor elements with infinite length. This ensured the applicability of image reconstruction methods that are based on strict two-dimensional wave propagation [8], [17]. To fulfill this requirement with sensor elements of finite length, a necessary condition is that any disturbance caused by the ends of the line sensors should not interfere with the signal arriving at normal incidence from anywhere in the object. Earlier we found a condition based on the assumption that the imaging object is touching the sensor [18]. However, for the current design we assumed that all objects should be confined within a sphere of radius *a* that is not touching the sensor line, as outlined in Fig. 2. From this sketch it follows that the minimum length of the line should fulfill the condition

(1) $$L=4\sqrt{a^{2}+aR}$$

The line sensors in the array were arranged on a semi-cylindrical surface with a radius of *R* = 50 mm. Assuming an imaging area with radius *a* = 20 mm centered on the axis of the cylinder led to a length of *L* = 150 mm. The array consisted of 64 sensor elements with a width of 1.5 mm, distributed on the cylindrical surface with an angular increment of 2.8°. For this number of elements we used a 32-channel data acquisition device with 2:1 multiplexing for each input.

### 2.2 Array manufacturing

**Fig. 3:**
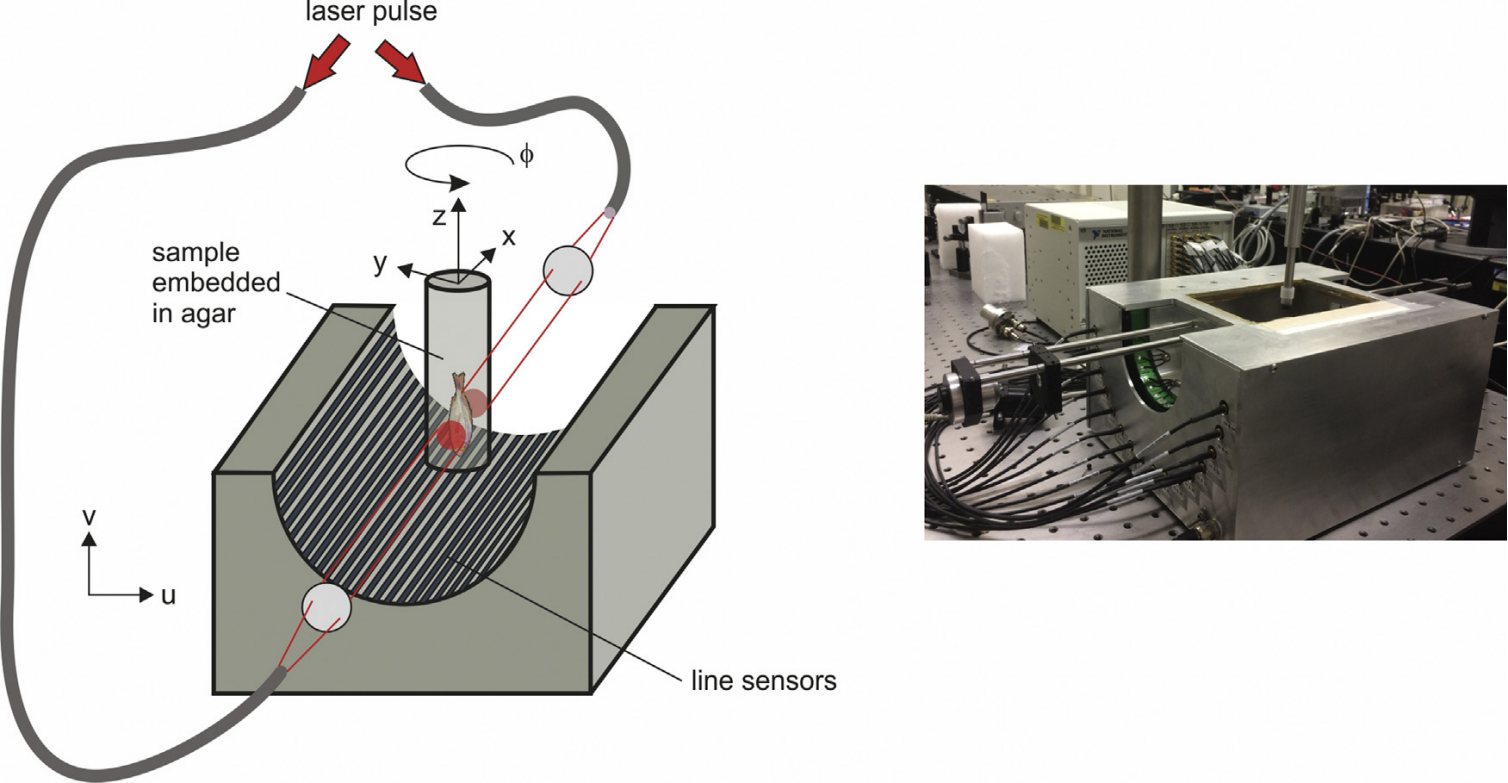
Drawing and photograph of the line detector tomograph. A sample, which may be fixed in agar, is inserted into the half cylindrical chamber of the tomograph that is filled with water. Laser pulses are delivered to the sample via glass fibers. The sketch also displays the coordinate systems used in the reconstruction: The coordinate system of the sample is (*x,y,z*). It is rotated relative to the tomograph by an angle *ϕ*. The coordinate system in the plane perpendicular to the direction of the line sensors in the tomograph is denoted by (*u,v*).

Fig. 3 shows a drawing of the tomograph. Copper electrodes with 35 μm thickness and the desired width and length formed the line pattern for the array on a semi-rigid FR4 printed circuit board of 1.5 mm thickness. The circuit board was glued onto a concave, half-cylindrical surface cut into a block of PVC. Onto the circuit board a sheet of 110 μm thick piezoelectric film of polyvinylidene fluoride (PVDF), (Measurement Specialties Inc.) was bonded. The film had a conductive, metallic coating on the upper side, forming the common ground electrode. Signals were picked up from the copper lines and led to 64 preamplifiers. Two preamplifier outputs were each multiplexed into one input of a second stage amplifier. Both amplifiers provided an overall gain of 59.5 dB in a bandwidth from 80 kHz to 40 MHz. The 32 outputs of the tomograph were connected to four analog-digital converter boards with eight inputs each (National Instruments PXI-5105), having a sampling rate of 60 MHz and a resolution of 12 bit.

Pulses from a Q-switched Nd:YAG laser at a wavelength of 532 nm pumped an optical parametric oscillator (OPO) (Innolas SpitLight OPO 600) at a repetition rate of 20 Hz. Two glass fibers with 1 mm core diameter guided the laser pulses to the tomograph, where they illuminated the sample from two opposite sides, parallel to the line detector orientation. Lenses imaged the fiber tips onto the sample, giving circular areas with adjustable size. Samples were held on a motorized rotation stage and submerged in water, which was filled into the half-cylindrical opening of the tomograph. A separate device provided the control signals for the multiplexer in the tomograph and the step motor in the rotation stage. In a Labview code that controlled the whole imaging procedure, the number of laser pulses over which the signals were averaged at each rotational position of the sample could be selected. Usually, the number of angular positions for a complete rotation of the sample was 400, with an increment of 0.9°. Since 2:1 multiplexing required two laser pulses at each position, the total number of laser pulses was 800 for acquiring data from a full rotation without signal averaging, taking 40 s.

### 2.3 Calibration

A calibration measurement was made in order to determine the relative sensitivities of the line sensors and their exact positions. These data were used for improving the accuracy of the image reconstruction. Although the tolerances in the manufacturing process were kept as low as possible, the radial distances of individual sensor elements relative to the cylinder axis varied slightly, due to small variations in the thickness of the adhesive layers between PVC substrate, circuit board and PVDF film. These were in the range of <100 μm, having an effect on the image resolution, which was expected to be on the same order of magnitude. For the calibration experiment, a small droplet of black oil paint with about 150 μm radius was embedded in a block of agar and was fixed near the axis of the cylindrical array. Due to the symmetric arrangement of illumination and sample, it could be expected that the amplitude of the spherical wave should be identical at all sensors. Variations occurring in the individual, detected amplitudes could therefore be attributed to the different sensitivities. To determine the exact radial positions of the sensor elements, the time of flight and travel distance of the spherical wave to each sensor element were extracted from the measured signals. Under the assumption that the deviations from the ideal cylindrical shape were small, a curve assuming constant radial position for all detectors was fitted to the experimental distances, yielding the exact source position and a value of the mean array radius. Adding the differences between the experimental and fitted distance values to the mean radius yielded the exact radial positions of the sensor elements.

### 2.4 Ultrasound response

The frequency response of the line sensors was simulated by calculating the response of the PVDF film to a plane wave at normal incidence. For this simulation we adapted a method used by Cox et al., who used it to calculate the frequency-dependent directivity of a planar polymer film used as Fabry-Perot interferometric ultrasound sensor [19]. This method gives also a response to waves at oblique incidence, but due to the cylindrical arrangement of the sensors and the confinement of the samples to a relatively small area around the cylinder axis, all waves were incident almost perpendicularly on the film. Therefore, we could reduce the analysis to normal incidence. In this simulation, water was assumed on top of the PVDF layer and a variable thickness epoxy adhesive layer followed by a copper substrate below. Since the simulations showed that the reflection at the upper copper surface had a strong effect, whereas the reflection at the copper – FR4 interface hardly influenced the signal, the copper electrode was assumed as infinitely extended in depth. This allowed us to simulate the sensor as a four-layer system. For comparison with an experiment, a phantom containing a black sphere with 100 μm diameter was irradiated along the direction of the line detectors. Due to the high absorption, the projection of the energy density distribution in the sphere into the *u,v*-plane gave a disk like source, for which we calculated the spectrum and multiplied it with the simulated transfer function of the layer system The result was then compared with the spectrum of the experimental signal. Since the electrical response was almost flat in the region of interest, it was not taken into account in this simulation.

### 2.5 Signal processing and image reconstruction

The image reconstruction procedure for line detectors has been described in detail earlier [8]. It will therefore only be briefly outlined here. Reconstructing a 3D image from measurements made with a line detector array is a two-step procedure. In the first step, signals measured at each angular position *ϕ* of the sample are used to reconstruct 2D images. Due to the integrating property of the line detectors, these images are projections of the initial pressure distribution in the sample into a plane spanned by coordinates *u* and *v* perpendicular to the line direction. This distribution is directly proportional to the absorbed energy density. To reconstruct a projection, an algorithm based on two-dimensional acoustic wave propagation is employed, where the recorded pressure signals are back projected onto cylinders with the line detector in the center. It can be derived from the universal back projection algorithm, which was initially established for three-dimensional photoacoustic tomography with point like detectors [8], [20]. Before back projection, the recorded signals are denoised by wavelet filtering or by applying a band pass filter. The back projection algorithm requires an exact value of the sound speed for conversion from the time axis to a distance from the sensor elements. Due to the temperature dependence of the sound speed it was therefore necessary to determine the exact temperature of the water bath in each experiment. Furthermore, the back projection requires the exact locations of the sensor elements, which were determined by the help of the calibration procedure outlined above.

**Fig. 4:**
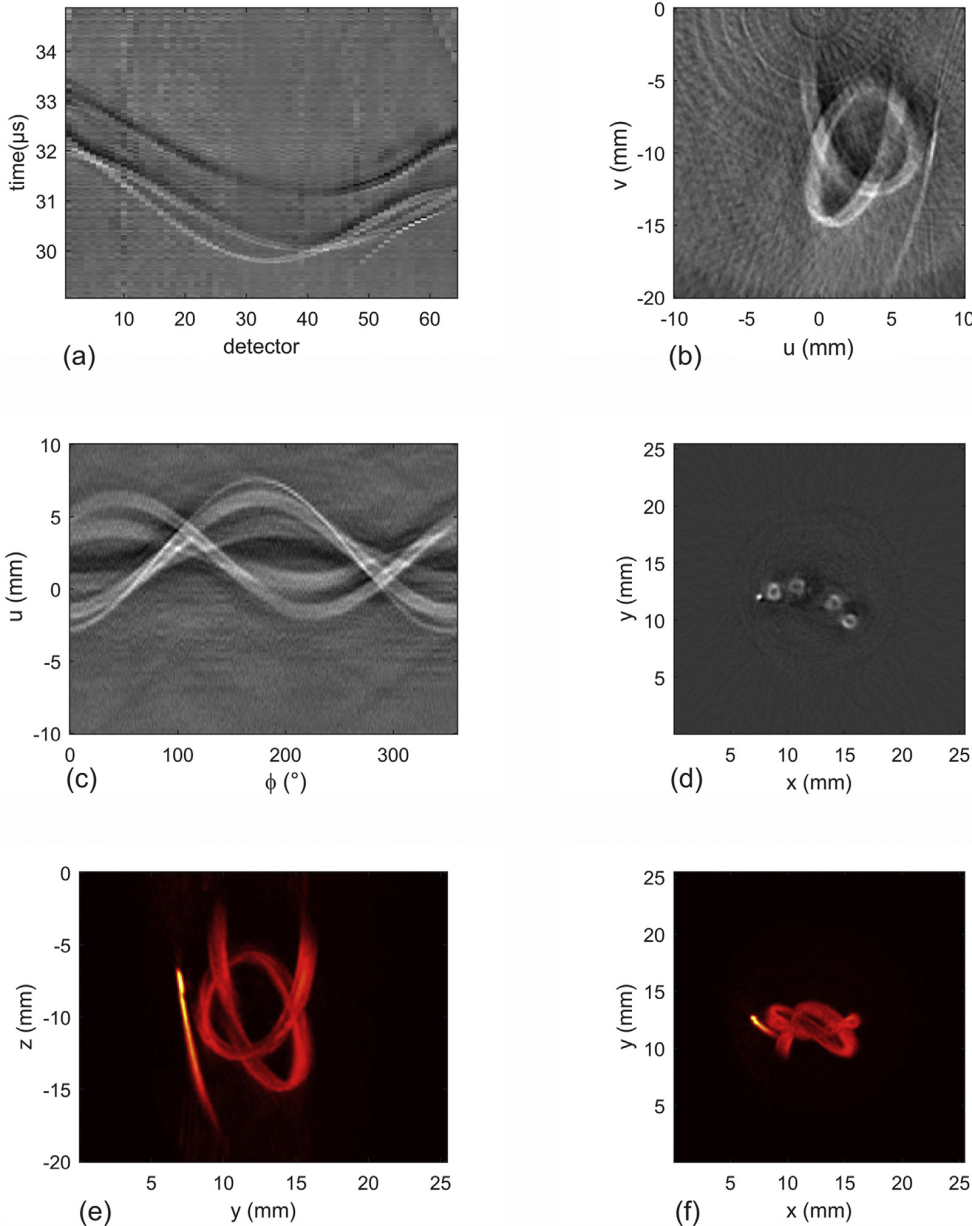
Imaging of a phantom consisting of a plastic tube filled with absorbing liquid in scattering agar. (a) Raw data showing time dependent pressure signals for each sensor. (b) Projection image generated from data in (a). (c) Data (sinogram) for one *v*-layer of the sample, taken from all projection images. (d) Section image reconstructed from the sinogram in (c) by an inverse Radon transform. (e), (f) Maximum amplitude projections of the 3D reconstruction.

By rotating the sample relative to the tomograph about an axis parallel to the *v*-direction, projections of the initial pressure distribution are recorded for *ϕ*-values covering at least 180°. For a complete 3D reconstruction, the final step is to apply the inverse Radon transform to projection data in planes with constant *v*, perpendicular to the rotation axis. This final step of the reconstruction requires the exact localization of the axis of the rotary stage relative to the reconstructed volume. Any slight movement of this axis relative to the detector array, for instance during mounting of the sample, could deteriorate the quality of the resulting image. Therefore, we implemented a method to determine the exact *u*-coordinate of the rotation axis from the recorded projection images. A small, photoacoustic source generates a sinusoidal structure in the input signals for the inverse Radon transform. These input signals are the line projections of the initial pressure distribution as a function of distance *u* and of the angle *ϕ*. They are the classical “sinograms” as they are found, for instance, in X-ray computed tomography with parallel beams. From such a sinusoidal curve generated by a point source the position of the rotation axis is found by determining its symmetry line. The steps of the reconstruction are illustrated in the next section, Fig. 4.

## 3 Results

### 3.1 Phantom image

A phantom image was acquired to demonstrate the steps from the initial data to the final 3D image. The phantom consisted of agar mixed with SMOFLipid, which contains soybean oil, similar to Intralipid. The mixture contained 0.6% fat. Embedded in the agar was a plastic tube with 1 mm inner diameter, which was filled with an indocyanine (ICG) solution (0.5 g/l) and was formed to a knot. Data were acquired at an OPO wavelength of 750 nm, where the absorption coefficient of the ICG solution was 140 cm^−1^. A pulse energy per fiber of about 6 mJ was used, giving a radiant exposure of ∼ 3 mJ/cm^2^ from each side. Fig. 4a shows the temporal pressure signals at a given orientation for all 64 detectors. From these signals a single projection image was reconstructed in the *u-v*-frame of the tomograph using the back projection algorithm (Fig. 4b). After completing the reconstruction of 2D projection images over the full angular range from 0° to 360°, the next step in the 3D reconstruction used the projection data of individual *v*-layers as input for the inverse Radon transform. Fig. 4c shows a sinogram for one specific *v*-layer, where the data are arranged as a function of angle *ϕ* and distance *u* to the center of the array. The corresponding 2D reconstruction is depicted in Fig. 4d. Finally, when all layers were reconstructed, the full 3D image was available. Two maximum amplitude projections of the 3D dataset are shown in Fig. 4e and f. Although the two-step procedure with back projection and subsequent inverse Radon transform seems to be rather time consuming, it requires only on the order of 2 * N^4^ operations for a data cube of size N^3^, where N is the number of reconstructed points along one direction in space. In this estimate it is assumed that signals are measured on an array with size N and that the number of orientations is also N. A back projection reconstruction in a volume of size N^3^ from data acquired at N^2^ detector positions on a plane would require on the order of N^5^ operations. Reconstructions were performed with Matlab codes on a standard PC. A single projection image was completed in 0.10 s. The whole 3D reconstruction from 400 projections, including the inverse Radon transform of 200 layers, took 130 s.

### 3.2 Characterization of the tomograph

#### 3.2.1 Calibration

**Fig. 5:**
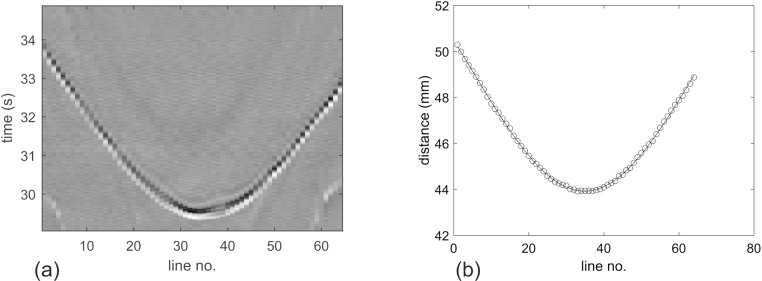
Calibration measurement. (a) Raw data obtained from a spherical photoacoustic source mounted about 6 mm below the center of curvature of the tomograph. (b) Arrival times (circles) of the spherical wave at the individual sensor elements and fitted smooth curve (solid line) assuming constant radial position of all lines.

**Fig. 6:**
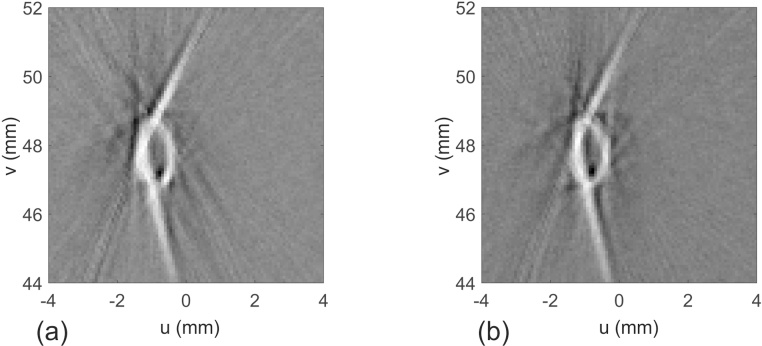
Projection image of a horsehair with a knot. (a) Reconstruction with corrections for variations in sensitivity and exact position of the detectors, (b) without corrections.

Fig. 5a shows the raw signals measured from the small, spherical source. The sphere was mounted slightly below the center of the array, where it could be fully illuminated by the laser pulses. Apart from the fluctuations in amplitude, there are also deviations in the arrival time from what would be expected for a perfect cylindrical arrangement of the sensors. The latter can be more easily seen in Fig. 5b, where a smooth curve assuming constant radius is fitted to the experimental distances. The deviations are within ±100 μm and were used to calculate the exact radial positions of the sensors, as described above. Fig. 6 shows a projection image of a horse hair with a knot, with and without corrections. Without correction, the line along the loop is not perfectly continuous. It turns out that the correction for variations in exact detector positions has more influence on the image quality than the correction of detector sensitivities.

#### 3.2.2 Resolution

**Fig. 7:**
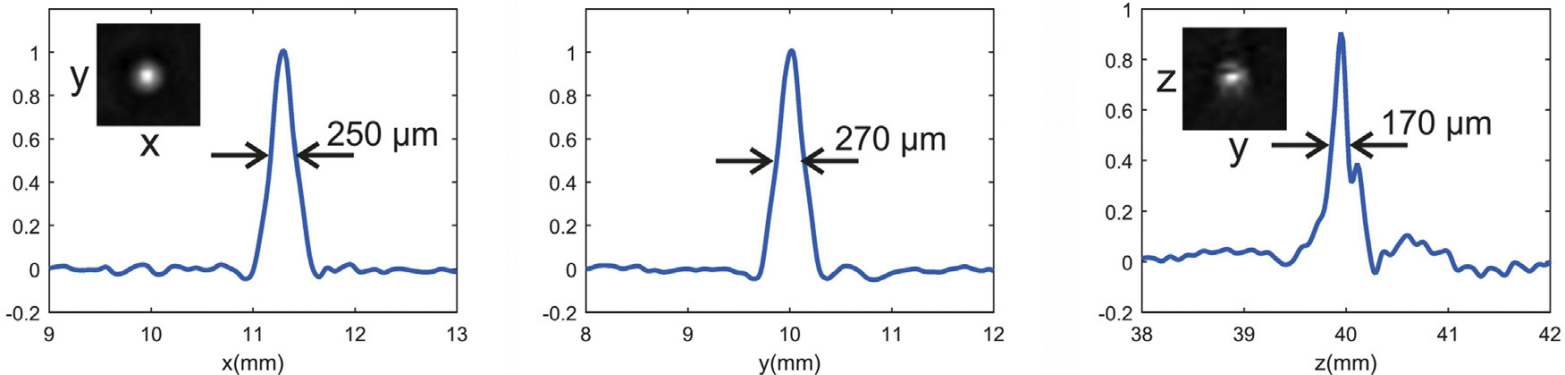
Resolution of the tomograph. Sections through the 3D image of a microsphere with 100 μm diameter (insets) were used to generate profiles in three directions in space. For each profile, the full width at half maximum is indicated.

To estimate the spatial resolution of the device, we built a phantom containing several black polystyrene microspheres with 100 μm diameter, embedded in scattering agar. Fig. 7 shows sections of the reconstructed 3D image and profiles through the center of a sphere in all directions in space. The width of the spheres in the profiles (FWHM) is approximately equal in all directions and lies between 170 and 270 μm. Considering the finite size of the spheres, this can be regarded as an upper limit of the resolution.

#### 3.2.3 Sensitivity

We made a measurement of the sensitivity of the line detectors using a phantom similar to the one shown above in Section 3.1. Again, tubes filled with an ICG solution were embedded in scattering agar. This time the tubes were oriented parallel to the line detectors and were irradiated by one optical fiber over a length of about 2 cm. OPO pulses with a radiant exposure of 2.6 mJ/cm^2^ at 800 nm wavelength were used. From Monte Carlo simulations using the optical constants of the phantom, we obtained the energy density deposited in the absorbing liquid (*μ_a_* = 8 cm^−1^) within the tubes. For the scattering coefficient of the agar, we used values of SMOFlipid (*μ_s_* = 11.5 cm^−1^, *g* = 0.5) reported by Held et al. [21]. The energy density distribution was used as the input for a simulation of sound propagation in an acoustically homogenous medium, yielding the expected amplitudes of the sound waves arriving at the detector lines. The experiments yielded the signal to noise ratio (SNR) for a tube signal. Dividing the simulated tube signal amplitudes by the experimental SNR gave a value for the pressure amplitude arriving at a detector that yields a signal equal to the noise level. The result was 20 Pa for a measurement without signal averaging. It has to be noted that due to the integrating effect of the line detectors the parallel arrangement of absorbing tubes and detectors gives rise to a higher SNR than any other orientation.

#### 3.2.4 Bandwidth

**Fig. 8:**
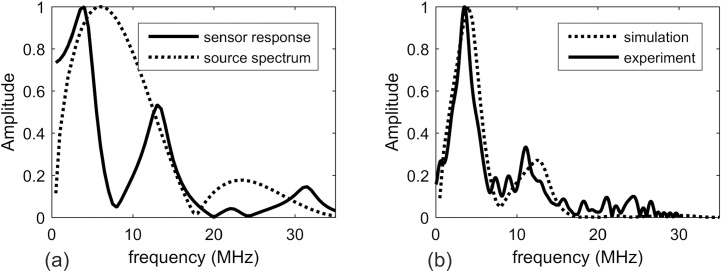
Bandwidth of the line transducers. (a) Calculated transfer function of the PVDF elements and spectrum of the signal from a disk-like source, calculated for an ideal transducer. (b) Spectra of simulated and experimental signals from a disk-like source.

Fig. 8a shows the simulated transfer function for a system consisting of the 110 μm thick PVDF film followed by a 10 μm layer of epoxy adhesive and the copper electrode. It is displayed together with the spectrum of the signal from the disk-like source. The adhesive layer thickness was derived by seeking the best match between simulation and experiment (Fig. 8b). In particular, the minimum near 8 MHz was sensitive to the layer thickness, since it is mainly due to the reflection of the wave at the copper electrode. Without the adhesive layer, this minimum appears at 10 MHz. The second minimum in the calculated transfer function at 20 MHz is determined by the thickness of the PVDF film.

### 3.3 In vivo imaging

**Fig. 9:**
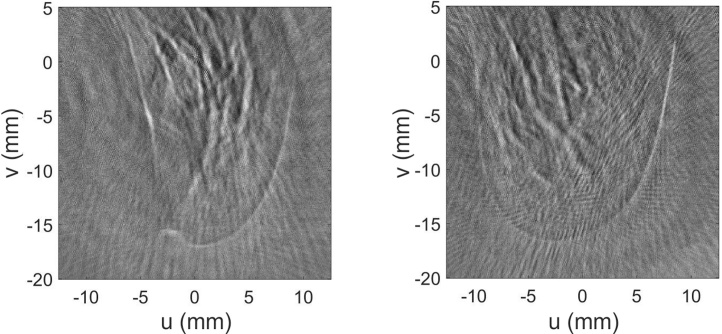
In vivo projection images of a human finger taken at a wavelength of 750 nm. Two perpendicular orientations of the finger are seen.

In vivo images of the fingertip of one of the authors were taken at a wavelength of 750 nm. The finger was fixed vertically (parallel to the *v*-axis) near the center of the cylindrical array and illuminated from one side along the direction of the cylinder axis with a radiant exposure of 16 mJ/cm^2^. In this experiment, free beam illumination was used instead of optical fibers, allowing a higher pulse energy at the target. With five times averaging and multiplexing, a single projection was captured in 0.5 s. Fig. 9 shows two projection images at two orientations of the finger differing by approximately 90°. Several features can be distinguished, mainly blood vessels, which are typical for in vivo photoacoustic images. In the side view also the nail bed is seen.

## 4 Discussion

In this work, we report on the design and manufacturing of a photoacoustic array tomograph capable of generating almost real-time 2D projections and 3D tomographic images of the absorbed energy distribution in biological objects. Projection images are generated because of the integrating effect of the extended line detectors in the array. They can be useful for the observation of objects in an extended volume. In the case of the line detector tomograph presented here, this volume is given by the length of the line sensors times the cross sectional area of the illuminating laser beam. An object located anywhere within this volume will be visible in the projection image. Fig. 4 shows a comparison between a directly measured projection image (b) and a maximum amplitude projection of the resulting 3D reconstruction (e). Although they look similar, the generation of the latter is a result of the complete 3D reconstruction, involving all detector orientations. It therefore has a much lower noise level than the direct projection image. However, the 2D projection image from the sensor data at a given orientation can be obtained almost in real time. Possible applications are the monitoring of dynamic processes or of procedures like needle biopsies, where a single or a small number of viewing angles yield already sufficient information to localize structures of interest.

An important step in the manufacturing of a PVDF array is the production and contacting of the sensor elements. PVDF film can be purchased in large sheets with metal electrode layers deposited on both sides. Separate elements can be created by patterning the electrodes, e.g. by etching. Especially for the long and thin line elements used in our study, there is always a risk that a thin scratch on the nanometer thin metal film disrupts the electrode, leading to the loss of entire elements. Therefore, we chose to use a method that had been proposed earlier for generating small, point like detectors. It uses metal electrodes on a circuit board adjacent to the piezoelectric film to determine the shape and extension of the sensor elements [22], [23]. This allows an easy integration of the sensors in an electronic circuit containing further signal conditioning devices such as amplifiers and multiplexers. A drawback of this design is a potential nonuniformity of the adhesive layer thickness. This gives rise to a variability of detector sensitivities and to small variations of radial detector positions. Both effects can be corrected by a calibration measurement with a small photoacoustic source. As the results in Fig. 6 show, the corrections cause only minute changes and can be omitted in most cases.

The design of the array was guided by several requirements. For instance, the array dimensions were chosen for a typical sample size of four centimeters. This determined the length of the lines (according to Eq. (1)) and also the radius of the array, since the best resolution in a circular detector arrangement can be expected for an area close to the center of the circle [7]. From this follows that for smaller or larger sample sizes the dimensions of the array can be changed accordingly. A number of 64 sensors was a compromise between the number of available channels in the data acquisition device (32) and the requirement to avoid excessive image acquisition times due to multiplexing. However, this turned out to be one drawback of the current design: the relatively low number of detectors led to streak artifacts as they are visible in some of the projection images. A higher detector number would therefore be desirable to reduce this kind of artifact in real-time imaging. If post-processing of images is an option, also special image reconstruction methods are capable of reducing streak artifacts, for instance methods that minimize the total variation [24].

## 5 Conclusions

The presented line detector array is capable of generating 2D photoacoustic projection images of extended volumes with a small number of sensor elements in almost real-time. Three-dimensional imaging is possible by rotating of the object relative to the array. In combination with the very flexible piezoelectric polymer film technology, a photoacoustic tomography device can be manufactured that exhibits a relatively low level of complexity, making it a cost-effective alternative to commercial devices.

## Conflict of interest

The authors declare that there are no conflicts of interest.
